# Expression of cardiovascular-related microRNAs is altered in L-arginine:glycine amidinotransferase deficient mice

**DOI:** 10.1038/s41598-022-08846-1

**Published:** 2022-03-24

**Authors:** Märit Jensen, Christian Müller, Norbert Hübner, Giannino Patone, Kathrin Saar, Chi-un Choe, Edzard Schwedhelm, Tanja Zeller

**Affiliations:** 1 Department of Neurology, University Medical Center Hamburg-Eppendorf, 20246 Hamburg, Germany; 2 University Center of Cardiovascular Science, Department of Cardiology, University Heart and Vascular Center Hamburg, University Medical Center Hamburg-Eppendorf, 20246 Hamburg, Germany; 3 German Centre for Cardiovascular Research (DZHK e.V.), Partner Site Hamburg/Kiel/Lübeck, Hamburg, Germany; 4 Cardiovascular and Metabolic Sciences, Max Delbrück Center for Molecular Medicine in the Helmholtz Association (MDC), Berlin, Germany; 5 Charité – Universitätsmedizin Berlin, Corporate Member of Freie Universität Berlin and Humboldt-Universität zu Berlin, Berlin, Germany; 6 German Centre for Cardiovascular Research (DZHK e.V.), Partner Site Berlin, Berlin, Germany; 7 Institute of Clinical Pharmacology and Toxicology, University Medical Center Hamburg-Eppendorf, 20246 Hamburg, Germany

**Keywords:** Cardiovascular genetics, miRNAs, Cardiovascular diseases

## Abstract

In humans and mice, L-arginine:glycine amidinotransferase (AGAT) and its metabolites homoarginine (hArg) and creatine have been linked to cardiovascular disease (CVD), specifically myocardial infarction (MI) and heart failure (HF). The underlying molecular and regulatory mechanisms, however, remain unclear. To identify potential pathways of cardiac AGAT metabolism, we sequenced microRNA (miRNA) in left ventricles of wild-type (wt) compared to AGAT-deficient (AGAT^-/-^) mice. Using literature search and validation by qPCR, we identified eight significantly regulated miRNAs in AGAT^-/-^ mice linked to atherosclerosis, MI and HF: miR-30b, miR-31, miR-130a, miR-135a, miR-148a, miR-204, miR-298, and let-7i. Analysis of Gene Expression Omnibus (GEO) data confirmed deregulation of these miRNAs in mouse models of MI and HF. Quantification of miRNA expression by qPCR in AGAT^-/-^ mice supplemented with creatine or hArg revealed that miR-30b, miR-31, miR-130a, miR-148a, and miR-204 were regulated by creatine, while miR-135a and miR-298 showed a trend of regulation by hArg. Finally, bioinformatics-based target prediction showed that numerous AGAT-dependent genes previously linked to CVD are likely to be regulated by the identified miRNAs. Taken together, AGAT deficiency and hArg/creatine supplementation are associated with cardiac miRNA expression which may influence cardiac (dys)function and CVD.

## Introduction

L-arginine:glycine amidinotransferase (AGAT; EC: 2.1.4.1) and its metabolites homoarginine (hArg) and creatine play an important role in cardiovascular disease (CVD) such as myocardial infarction (MI), heart failure (HF) and ischemic stroke^1–5^. In clinical studies, lower hArg levels were associated with incident major adverse cardiovascular events (MACE) after acute coronary syndrome^3^ and all-cause mortality in patients with HF ^4^. Besides the prognostic value, experimental data have suggested a causal role of AGAT and its metabolites in CVD. AGAT deficiency in mice was found to be associated with an impaired cardiac function which was rescued by hArg supplementation and, to a lesser extent, by creatine supplementation^6^. Moreover, hArg has been shown to be cardioprotective in a model of ischemic HF and calcified coronary artery disease ^7,8^.

The underlying mechanisms are still unclear. In a previous transcriptome analysis in AGAT-deficient (AGAT^-/-^) mice, we identified significantly regulated genes between AGAT^-/-^ and wild-type (wt) mice that are involved in cardiac pathophysiology. These comprised genes affecting cardiac energy metabolism, cardiac hypertrophy and fibrosis, immune response, and the conduction system of the heart. Creatine rather than hArg supplementation in AGAT^-/-^ mice resulted in a restoration of gene expression towards wt levels^9^.

Non-coding RNAs such as microRNAs (miRNAs) have been shown to be effective modulators of gene expression, expanding the spectrum of molecular mechanisms controlling physiological and pathological cellular functions. Growing evidence suggests that miRNAs are pivotal regulators in CVD^10,11^. In experimental animal models of MI and HF, differential patterns of miRNA expression have been reported^12^. Therefore, the identification of miRNA expression patterns with cardiac phenotypic changes may help in understanding the underlying molecular mechanisms and pathways. AGAT^-/-^ mice have been well-characterized regarding cardiovascular changes on phenotype and on gene expression level, but no data on AGAT-dependent cardiac miRNA expression is available.

In the present study, we aimed to expand the understanding of underlying molecular (patho)mechanisms of AGAT and its metabolites in CVD. To this end, we used a previously established AGAT^-/-^ mouse model^13^ to characterize cardiac miRNAs signatures within the AGAT metabolism. Our first objective was to study AGAT-dependent regulation of miRNAs and their association to CVD, specifically atherosclerosis, MI, and post-MI HF. Secondly, we aimed to determine whether AGAT-dependent changes in miRNA expression are related to either hArg or creatine. Third, we studied miRNA-mRNA interactions in our AGAT^-/-^ mouse model to identify regulatory mechanisms within the cardiac AGAT metabolism.

## Methods

### Study design

**Figure 1:**
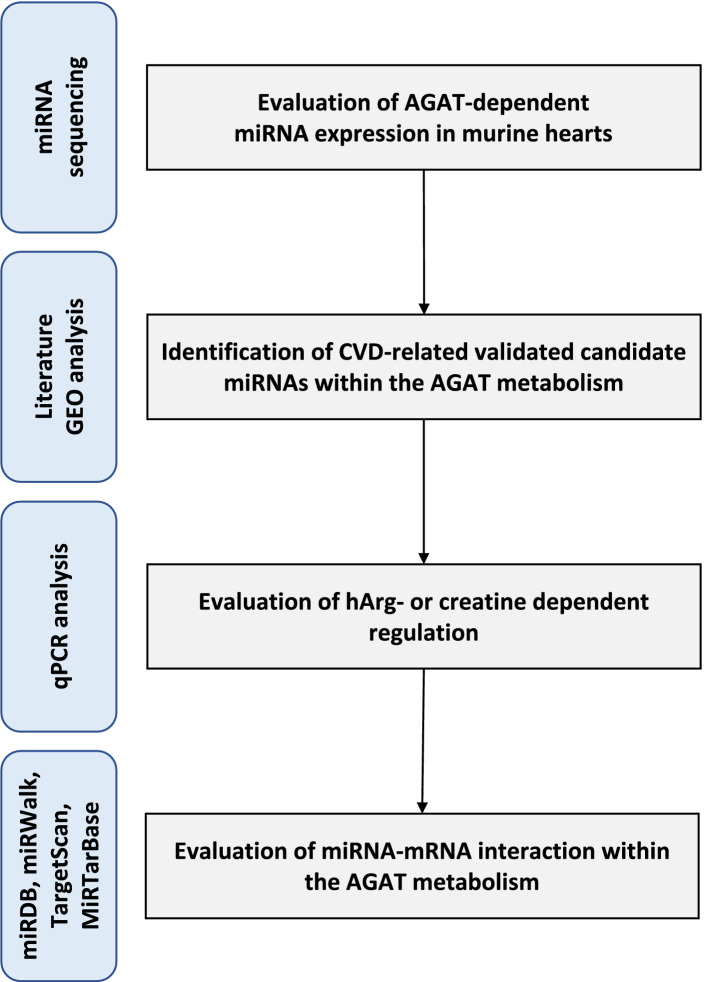
Overview of the methods and objectives of the different steps of the study design. CVD: cardiovascular disease; GEO: Gene Expression Omnibus; hArg: homoarginine; miRNA: microRNA.

Our study design followed a sequential approach (Fig. 1). First, we performed miRNA sequencing in heart tissue of AGAT^-/-^ as compared to wt mice. Second, we checked whether identified miRNAs were already reported to be associated with CVD by a literature search in PubMed/MEDLINE (search terms: miRNA of interest and “cardiac” or “heart” or “cardiovascular disease”). Of these miRNAs only those that could be validated by qPCR were used for further analysis. To test the role of candidate miRNAs in mouse models of CVD, we performed Gene Expression Omnibus (GEO)^14^ data analysis. Third, we evaluated whether miRNA expression was dependent on hArg or creatine by analysis in AGAT^-/-^ mice supplemented with either hArg or creatine. Finally, we aligned miRNAs to AGAT-dependent mRNAs identified by microarray analysis and tested whether these genes were known to be involved in CVD.

### Care and treatment of mice

AGAT^-/-^ mice were generated as previously described^13^. Mice used in this study were obtained from heterozygous breeding after backcrossing to a C57BL/6 J genetic background for at least six generations. All analyzed animals were littermates. The mice (< 5 per cage) were kept in standard cages under a 12 h:12 h light:dark cycle and constant temperature and humidity, receiving standard food and water ad libitum. The 4-week-long supplementation with hArg was achieved via osmotic mini pumps^15^. Creatine supplementation was achieved by addition of 1% creatine to chow (Ssniff)^13^. Plasma and tissue concentrations of creatine and hArg in wt and AGAT^-/-^ mice have been decribed previously^13,15^. All experimental procedures were approved by the respective local animal ethics committees (Behörde für Gesundheit und Verbraucherschutz Hamburg, approval no. 110/10, approval date January 31, 2011) and investigations applied to the animal model were in accordance with the guidelines for the care and use of laboratory animals published by the NIH (Publication No. 85–23, revised 1985). The study was carried out in compliance with the ARRIVE guidelines^16^.

### Tissue collection and preparation

Tissue collection and preparation was performed as previously described^13^. Briefly, mice were anesthetized with 2–3% isoflurane in 100% oxygen. Left ventricles (LV) of the heart were extracted, and snap-frozen in liquid nitrogen for storage at − 80 °C. Prior to use, frozen tissue was powdered with a steel mortar and pestle in liquid nitrogen.

### RNA isolation from murine heart tissue

Total RNA, including the miRNA fraction, was prepared in four groups of mice: wt (n = 7), AGAT^-/-^ (n = 7), AGAT^-/-^hArg (n = 5) mice and AGAT^-/-^ mice supplemented with creatine (AGAT^-/-^Cr, n = 4). Isolation was performed using QIAzol lysis reagent (QIAGEN). RNA integrity and the content of miRNAs was determined using the Agilent 2100 Bioanalyzer (RNA 600 Nano Chip, Agilent Small RNA Chip).

### MiRNA sequencing

MiRNA sequencing was performed in wt (n = 5), AGAT^-/-^ (n = 5), and AGAT^-/-^hArg (n = 5) mice. Small RNA libraries were prepared using TruSeq Small RNA Sample Preparation Kits (Illumina, San Diego, CA, USA) according to the manufacturer’s instructions. In brief, 3’ and 5’ RNA adapter, specifically modified to target the ends of miRNA molecules, were ligated to 1 μg of high-quality total RNA. Reverse transcription was used to generate single-stranded cDNA libraries and PCR was performed to amplify and add unique index sequences to each library. MiRNA profiles were generated by deep sequencing using Illumina HiSeq 2500 sequencer.

### Gene expression profiling

Gene expression profiling was performed in wt (n = 7), AGAT^-/-^ (n = 7), AGAT^-/-^hArg (n = 5) and AGAT^-/-^Cr (n = 4) mice using the Affymetrix Mouse GeneChip ST 1.0 Array as described previously^9^. All groups of mice were analyzed at the same time. Briefly, cRNA synthesis, labelling, fragmentation, array hybridization, washing and staining, and microarray scanning (Affymetrix GeneChip 3000 scanner) was performed according to manufacturer’s instruction of the Ambion WT Expression Kit and the Affymetrix GeneChip WT Terminal Labelling and Hybridization Kit with an input of 250 ng high quality RNA (RNA integrity number > 8).

### Reverse transcription and quantitative real-time polymerase chain reaction

To validate expression levels of selected miRNAs in all groups of mice (wt, AGAT^-/-^, AGAT^-/-^hArg, AGAT^-/-^Cr), quantitative real-time polymerase chain reaction (RT-qPCR) was carried out using the Taqman MicroRNA assay (Thermo Fisher Scientific) according to manufacturer's recommendations. The TaqMan IDs for the miRNAs are shown in Supplementary Table S1. The human assay (‘hsa’) was used for miRNAs with homologous sequences among mice and humans. As murine miR-298 and miR-31 differ from the human sequence, a specific mouse assay (‘mmu’) was used for these miRNAs. All reactions were performed using a 7900 TaqMan system (Applied Biosystems, Foster City, CA, USA). Each sample was analyzed in triplicates and normalized to snoRNA202 as endogenous control.

### Analysis of miRNA expression in cardiovascular disease using Gene Expression Omnibus (GEO)

To evaluate expression profiles of selected miRNAs in CVD a systematic search in the GEO database^14^ was carried out with the following keywords: “miRNA” AND “heart” AND “Expression profiling by high throughput sequencing” for study type AND “tissue” for attribute name AND “mus musculus” for organism.

The GEO series GSE114695 was selected for in silico analysis, which is a time series (1 day, 1 week, and 8 weeks) intended to compare non-coding RNA expression in normal functioning LV with infarcted LV in C57BL/6 mice^17^. MI was mimicked by permanent ligation of the left anterior descending coronary artery in 8 weeks C57BL/6 male mice. Small RNA profiles were generated by deep sequencing using Illumina HiSeq 2000 sequencer. The reads from small RNA sequencing were aligned to mus musculus matured and precursor miRNAs obtained from miRBase v21 using the miRDeep2 algorithm. Read count data were downloaded and differential miRNA expression between hearts of MI and sham animals was calculated for each time point separately using R/Bioconductor’s DESeq2 package^18^.

The GEO series GSE112054 was selected for analysis of miRNA expression in HF which was induced by transverse aortic constriction (TAC) surgery in C57Bl/6 J mice^19^. At 5 weeks after surgery, mice were sacrificed, and LV myocardium was collected. MiRNA sequencing was performed on an Illumina HiSeq 2500. Data were analyzed using the miRge program (v1.0), which utilizes CutAdapt 1.8 to pre-process sequencing data and Bowtie 1.1.1 to perform read alignments, to generate miRNA counts and reads-per-million data. Read count data were downloaded and tested for differential miRNA expression between TAC and sham animals using DESeq2^18^.

### MiRNA target prediction tools

We used miRDB^20,21^, miRWalk^22^, TargetScan^23^, and miRTarBase^24^, which are freely available online, to predict potential miRNA target genes within the AGAT metabolism. Detailed information on the individual databases and criteria used to define relevant miRNA-mRNA interactions are given in Table 1.

**Table 1:** MiRNA target prediction tools.

	miRDB^20,21^	miRWalk^22^	TargetScan^23^	miRTarBase^24^
Website	http://mirdb.org	http://mirwalk.umm.uni-heidelberg.de	http://www.targetscan.org	https://mirtarbase.cuhk.edu.cn
Version	miRDB 2020	miRWalk2.0	TargetScanMouse 8.0	miRTarBase 9.0
Prediction criteria	Target prediction score > 80	Binding P value > 0.9	Conserved sites	Experimentally validated miRNA-mRNA interactions

### Bioinformatics analysis

The CLC Genomics Workbench (clcbio.com/products/clc-genomics-workbench/) was used to map reads from miRNA sequencing against the murine set of all known miRNAs, which was retrieved from miRBase (www.mirbase.org/). The number of reads falling in mature miRNAs were extracted and further processed in R. Only miRNAs covered by more than ten reads were kept for further analyses. Differential expression of miRNAs between groups of mice was calculated by R/Bioconductor package DESeq2^18^ and the False Discovery Rate (FDR) based Benjamini–Hochberg method was used to account for multiple tests. Differentially expressed miRNAs with an FDR ≤ 0.05 were considered significant.

Methods of differential gene expression analysis of microarray-based murine transcriptomes are described in detail in our previous publication^9^. The detection above background (DABG) was calculated for all genes and samples, and only genes with a DABG P value < 0.01 in at least two samples per group were kept for further analysis. Differential gene expression between groups was calculated using the moderated t-test function *eBayes* from the R/Bioconductor package *limma*^25^ and an FDR of ≤ 0.05 was set for statistical significance.

### Statistical analysis

Values are expressed as mean ± SEM. Expression levels were quantified according to the 2^-∆∆Ct^ method by Livak and Schmittgen^26^. Statistical comparison of more than two groups was performed using the Kruskal–Wallis test followed by Dunn’s test. Differences were considered statistically significant at a value of P < 0.05. All calculations were performed using GraphPad Prism (version 9, La Jolla, USA).

## Results

### Differences in miRNA expression in heart samples between wt and AGAT^-/-^ mice

**Figure 2:**
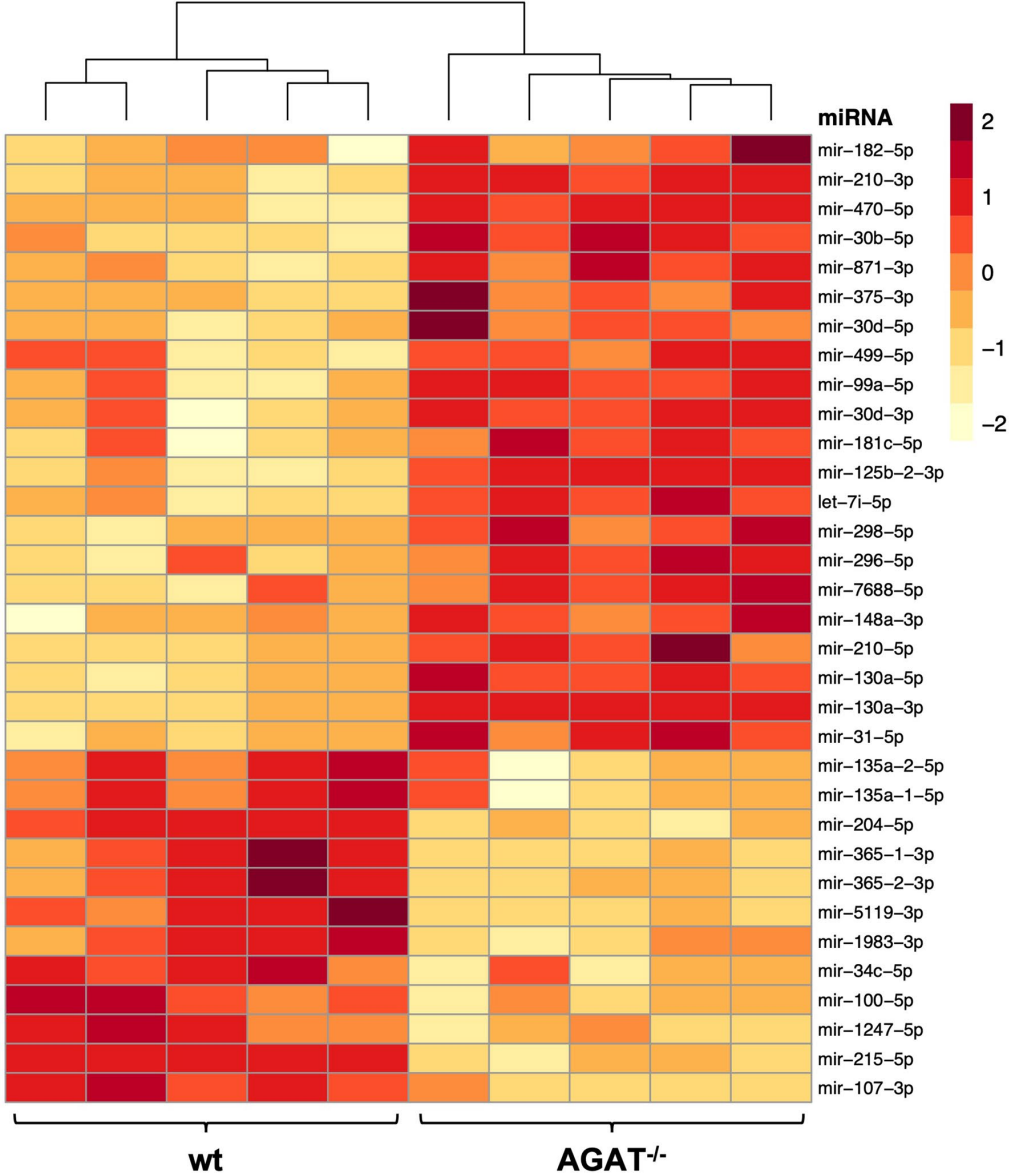
Significantly regulated miRNAs between wt and AGAT^-/-^ mice. Z score transformed heat map of miRNA sequencing results on AGAT^-/-^ in comparison with wt mice (n = 5 per group). Low to high expression is represented by a change of color from yellow to red. 3p and 5p indicates the origin of the miRNA from the 3' arm or 5' arm, respectively. Significance level: False Discovery Rate (FDR) ≤ 0.05. wt: wild type; AGAT^-/-^: AGAT knockout.

To determine the influence of AGAT deficiency on cardiac miRNA levels, we performed miRNA sequencing on LV from wt and AGAT^-/-^ mice. Among 953 miRNAs detected in LV samples, 33 showed significant regulation between wt and AGAT^-/-^ mice (Fig. 2, Supplementary Table S2). Quantification revealed that 21 miRNAs were upregulated in AGAT^-/-^ mice as compared with wt mice, while expression levels of twelve miRNAs were decreased.

### MiRNAs deregulated in AGAT^-/-^ hearts are linked to cardiovascular disease

**Figure 3:**
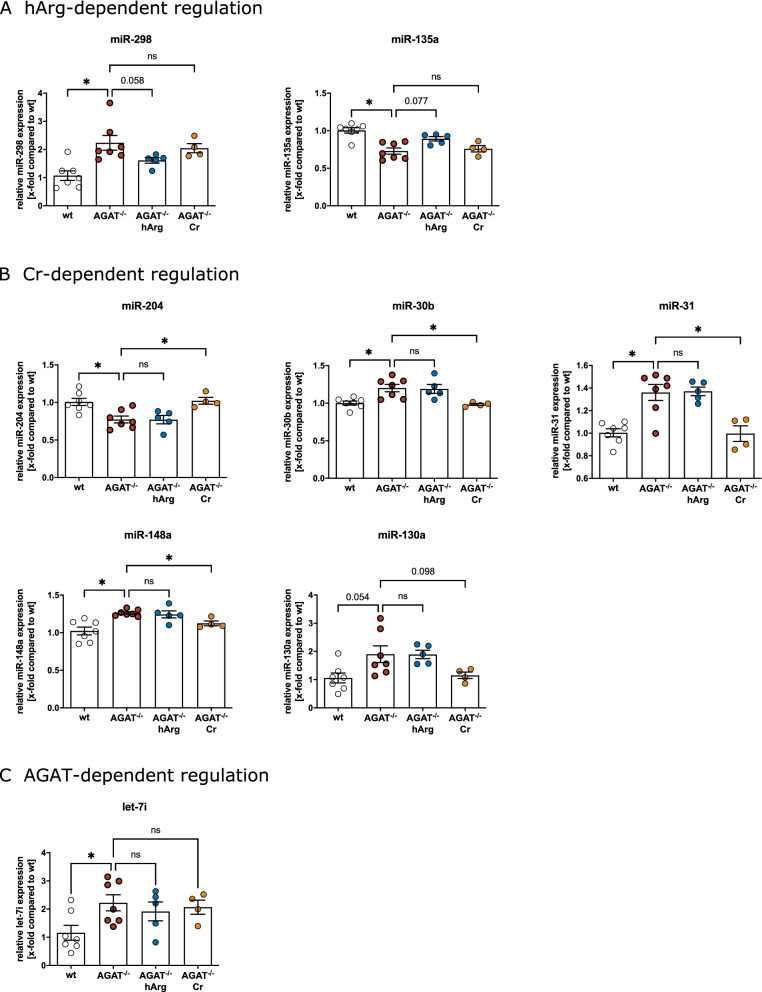
Analysis of candidate miRNAs in the heart by qPCR. Relative miRNA expression was measured in wild-type (wt; n = 7), AGAT knockout (AGAT^-/-^; n = 7), homoarginine-supplemented AGAT^-/-^ (AGAT^-/-^hArg; n = 5) and creatine-supplemented AGAT^-/-^(AGAT^-/-^Cr; n = 4) mice. Only miRNAs with positive validation are shown. Values are expressed as mean ± SEM. *P < 0.05 versus AGAT^-/-^.

We focused our analysis on miRNAs which were reported to be linked to CVD in multiple studies of either atherosclerosis, MI and HF, and could be validated by qPCR. Twelve out of 33 miRNAs deregulated in hearts of AGAT^-/-^ mice have been linked to CVD in previous studies, eight of which we could validate by qPCR. Table 2 presents the fold change for these eight differentially expressed miRNAs linked to CVD between wt and AGAT^-/-^ mice in heart tissue. Figure 3 A-C shows the results of qPCR analysis (wt vs. AGAT^-/-^ mice). MiR-130a was upregulated, while miR-204 was downregulated in AGAT^-/-^ mice. Both miRNAs are associated with vascular pathologies, particularly calcification of vascular smooth muscle cells and atherosclerosis^27–29^. Mir-298, miR-30b, and miR-31 were upregulated in AGAT^-/-^ mice and regulate cardiomyocyte apoptosis and cardiac remodeling after myocardial injury in vivo^30–32^. MiR-135a which was downregulated in AGAT^-/-^ as compared to wt animals has also been investigated with regard to myocardial ischemia and regulating cardiac fibrosis in HF^33,34^. MiRNAs from the let-7 family were upregulated in AGAT^-/-^ mice, and aberrant expression of let-7 members was found in various cardiac diseases with let-7i suspected to be a biomarker for dilated cardiomyopathy^35,36^. MiR-148a which was upregulated in response to the AGAT^-/-^ showed cardioprotective effects in HF^37^.

**Table 2:** CVD-related candidate miRNAs in murine heart tissue. FC and P values for differentially expressed miRNAs between wild-type and AGAT knock-out mice in heart tissue are shown. MiRNAs were selected based on known association with CVD and grouped according to the most prominent association with a specific CVD or risk factor. Relevant references are provided for each miRNA. FC: fold change.

Myocardial injury/infarction			Heart failure			Atherosclerosis		
miRNA	FC	P value	miRNA	FC	P value	miRNA	FC	P value
miR-298^30^	2.1	1.2 × 10^–7^	miR-148a^37^	1.23	7.6 × 10^–4^	miR-130a^27^	1.66	1.9 × 10^–29^
miR-135a^33,34^	− 1.63	2.7 × 10^–4^	let-7i^35,36^	1.16	3.7 × 10^–5^	miR-204^28^	− 1.22	1.4 × 10^–8^
miR-31^32^	1.5	6.4 × 10^–10^						
miR-30b^31^	1.2	3.8 × 10^–6^

### Candidate miRNAs are deregulated in mouse models of cardiovascular disease

**Figure 4:**
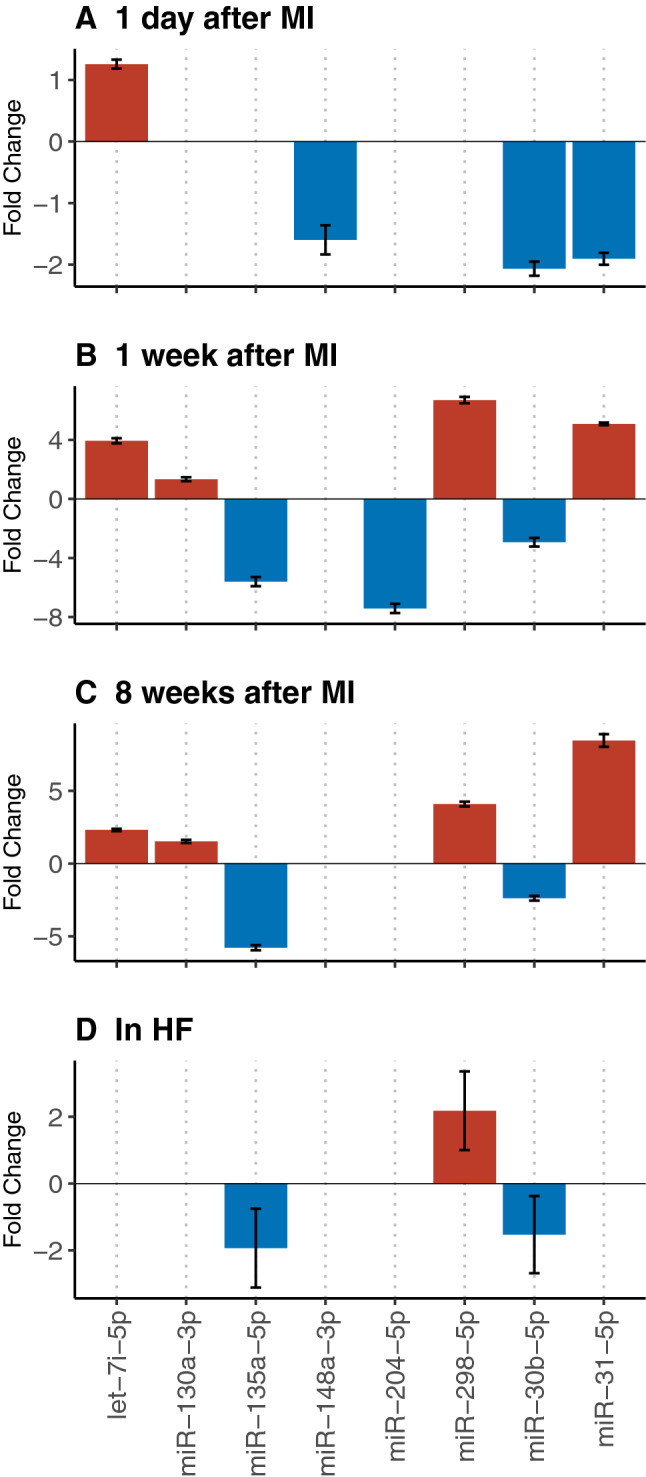
Expression of candidate miRNAs in mouse models of myocardial infarction (MI) and heart failure (HF). (**A**-**C**), Regulation of candidate miRNAs at 1 day, 1 week, and 8 weeks post MI. MI was induced by permanent ligation of the left anterior descending coronary artery in 8 weeks C57BL/6 male mice. Left ventricles were used for miRNA sequencing (GEO accession GSE114695). (**D**) Regulation of candidate miRNAs in HF. HF was induced by transverse aortic constriction (TAC) surgery in C57Bl/6 J mice. At 5 weeks after surgery, mice were sacrificed, and LV myocardium was used for miRNA sequencing (GEO accession GSE112054). Bars indicate fold changes in miRNA expression in MI or HF as compared to wt mice. Bars are only shown when significant regulation was observed. 3p and 5p indicates the origin of the miRNA from the 3' arm or 5' arm, respectively.

In order to investigate regulation of our candidate miRNAs in in vivo mouse models of CVD, we systematically searched for experimental data in the GEO Profiles database^14^. We identified two studies with available miRNA deep sequencing data on MI (GEO accession GSE114695^17^) and HF (GEO accession GSE112054^19^) in C57BI/6 J wt mice. As illustrated in Fig. 4A-C, all candidate miRNAs showed regulation in response to experimentally induced MI, which was particularly pronounced one week after MI. Eight weeks after MI which comes along with the development of post-MI HF in mice, we found that six out of eight candidates were differentially expressed in diseased as compared to healthy mice. Another study on HF using TAC surgery, which is a common experimental model for pressure overload-induced cardiac hypertrophy and heart failure, revealed decreased expression levels of miR-30b and miR-135a, while miR-298 was upregulated (Fig. 4D).

### Analysis of homoarginine- and creatine dependence of miRNA regulation in the heart

Creatine and hArg supplementation have beneficial effects in AGAT^-/-^ mice on heart function and outcome in CVD, respectively. Therefore, we aimed to evaluate whether creatine or hArg supplementation regulates candidate miRNA expression in hearts of AGAT^-/-^ mice. In our study miRNA sequencing data were only available for wt, AGAT^-/-^, and AGAT^-/-^hArg mice. Using qPCR analysis, we additionally included the group of creatine-supplemented animals. As compared to AGAT^-/-^ mice, the group of AGAT^-/-^hArg littermates showed a trend towards decreased expression of miR-135a (P = 0.077) and miR-298 (P = 0.058). Levels of these miRNAs were restored towards wt levels suggesting a hArg-dependent regulation (Fig. 3A). In AGAT^-/-^Cr as compared to AGAT^-/-^ mice, miR-30b, miR-31, miR-148a, and miR-204 showed significant regulation restoring miRNA expression levels towards wt levels. For miR-130a, a trend in the same direction was observed (P = 0.098) (Fig. 3B).

### MiRNA-mRNA interactions within the AGAT metabolism

To identify potential regulatory mechanisms within the AGAT metabolism, we combined miRNA and mRNA data of our AGAT^-/-^ mouse model using the bioinformatics-based target prediction tools miRDB^20,21^, miRWalk^22^, and TargetScan^23^ as well as the experimentally validated miRNA target interactions database miRTarBase^24^. We restricted the analysis to the eight validated candidate miRNAs and 485 differentially expressed mRNAs between wt and AGAT^-/-^ mice which we reported previously^9^. The results revealed several AGAT-dependent genes described to be associated with CVD as potential targets for regulation by candidate miRNAs (Table 3). We identified 17 target genes of the eight candidate miRNAs, one of which was targeted by two miRNAs. For seven of overall 17 potential target genes, we found studies reporting a role in CVD, comprising HF and cardiac remodeling after MI (*Ccnd2*, *Pde1c*, *Ddah1*), atherosclerosis (*Igf1r*), blood pressure and hypertension (*Fign*, *Efnb3*), and type 2 diabetes (*Igf2bp2*).

**Table 3:** Potential mRNA targets of candidate miRNAs within the AGAT metabolism. The analysis was performed using the online tools miRDB, miRWalk, TargetScan, and miRTarBase. For the individual databases, interactions were considered relevant when the following criteria were met: miRDB – target prediction score > 80; miRWalk – binding p-value > 0.9; TargetScan – conserved sites; miRTarBase – experimentally validated targets. All interactions which were predicted in at least three out of four of the online tools are listed in the table. CVD: cardiovascular disease; MI: myocardial infarction; n.a.: not applicable (no association with CVD reported).

miRNA	Target gene	miRWalk	TargetScan	miRDB	miRTarBase	Role in CVD
let-7i-5p	*Ccnd2*	X	X	X		Recovery after MI ^45^
	*Fign*	X	X	X		Blood pressure and arterial hypertension^46^
	*Igf2bp2*	X	X	X		Type 2 diabetes, physiology of cardiomyocytes^47^
miR-130a-3p	*Memo1*	X	X	X		n.a
miR-148a-3p	*Phactr2*	X	X	X		n.a
miR-204-5p	*Efnb3*	X	X	X		Blood pressure regulation^48^
	*Ezr*	X	X	X		n.a
	*Chp1*	X	X	X		n.a
	*Ociad2*	X	X	X		n.a
	*Pde1c*	X	X	X		Heart failure^49^
	*Fam126a*	X	X	X		n.a
miR-298-5p	*Igf1r*	X		X	X	Atherosclerosis^50^
miR-30b-5p	*Lgi1*	X	X			n.a
	*Fign*	X	X	X		Blood pressure and arterial hypertension^46^
	*Ddah1*	X	X	X		Cardiac remodeling after MI^51^
miR-31-5p	*Bahd1*	X	X	X		n.a
miR-135a-5p	*Prlr*	X	X	X		n.a
	*Zfp385b*	X	X	X		n.a

## Discussion

In this study, we aimed to better understand potential molecular mechanisms underlying the association of AGAT and its metabolites hArg and creatine with cardiac function and CVD. To this end, we investigated the effect of AGAT-, hArg- and creatine deficiency on cardiac miRNA expression. As our first main result, eight validated AGAT-dependent miRNAs were associated with CVD in both literature and in silico analysis of murine MI and HF experiments (miR-30b, miR-31, miR-130a, miR-135a, miR-148a, miR-204, miR-298, let-7i). Second, miR-30b, miR-31, miR-130a, miR-148a, and miR-204 were regulated by creatine, while miR-135a and miR-298 showed a trend of regulation by hArg. Third, candidate miRNAs were predicted to regulate numerous AGAT-dependent genes previously reported to be associated with CVD pathways.

There is ample evidence from clinical and experimental studies supporting the link between AGAT and its metabolites and CVD^1–4,6,7^. Both creatine and hArg are prognostic markers for CVD^1–5^. In the heart, creatine acts as a rapidly available energy buffer through its involvement in the creatine kinase (CK) system^38^. Most of the components of this CK system are downregulated in HF, with levels of creatine, phosphocreatine, and relevant CK isoforms all greatly reduced in animal models and humans^39,40^. However, the exact contribution of reduced creatine to cardiac pathophysiology remains controversial. The physiological role of hArg is even less understood. Given its structural similarity to L-arginine, hArg can serve as an alternative substrate for nitric oxide synthase and, in support of this, hArg levels have been linked to endothelial function^41^. Furthermore, hArg can competitively inhibit arginase, thereby increasing the bioavailability of L-arginine and subsequently nitric oxide production^41^. In epidemiological studies hArg levels were inversely associated with aortic wall thickness, aortic plaque burden and internal carotid artery stenosis^42–44^ suggesting an involvement in atherosclerosis.

We used a previously developed AGAT^-/-^ mouse model with hArg and creatine deficiency which has been well-characterized regarding metabolic changes and organ dysfunction^13^. The cardiovascular phenotype of these AGAT^-/-^ mice is characterized by low LV end-systolic pressure (LVESP), impaired contractility and relaxation, as well as an altered response to dobutamine infusion compared to wt mice^6^. In a previous transcriptome analysis of LV tissue in this AGAT^-/-^ mouse model, we identified differences of gene expression between AGAT^-/-^ and wt mice, affecting cardiac energy metabolism, cardiac hypertrophy and fibrosis, immune response, and the conduction system of the heart.

Non-coding RNAs such as miRNAs are a promising target for cardiovascular research, as they are involved in regulation of gene expression and have diagnostic and prognostic value in CVD^10,11^. Here, we performed miRNA sequencing and found eight validated CVD-related miRNAs to be deregulated in AGAT^-/-^ as compared to wt mice. Amongst these, miR-130a and miR-204 were previously described to be associated with atherosclerosis, miR-30b, miR-31, miR-135a, and miR-298 with MI, and miR-148a and let-7i with HF. The potential role of the candidate miRNAs in the pathophysiology of CVD is further supported by the results of GEO miRNA expression analysis in mouse models of MI and HF. We were able to show a deregulation of all our candidates in CVD. Based on these results, we suggest that an impaired molecular adaptability via miRNAs in altered AGAT metabolism may contribute to the association of hArg and creatine with CVD.

Both hArg and creatine may underlie the association of AGAT deficiency with deregulation of CVD-related miRNAs. Experimental data suggest that especially hArg plays a major role regarding impaired cardiac function in AGAT^-/-^ mice but also may be protective in CVD in wt mice. Impaired cardiac function in our AGAT^-/-^ mouse model was rescued across several parameters by hArg supplementation, while creatine supplementation only corrected LVESP^6^. In a model of post-MI HF, hArg-supplemented wt mice showed a normalization of several cardiac parameters (i.e., relaxation, cardiac reserve)^7^. In our study, we observed a trend of restored miRNA expression of miR-135a and miR-298 towards wt levels after supplementation of hArg in AGAT^-/-^ mice. Experimental studies revealed that deregulation of miR-298 may be involved in regulating apoptosis of cardiomyocytes after myocardial injury^30^. Interestingly, miR-135a, which was downregulated in AGAT^-/-^mice, was reported to inhibit myocardial fibrosis, a common hallmark in various heart diseases such as HF after MI^34^. This regulation was mediated via targeting the transient receptor potential cation channel subfamily M member 7 (TRPM7) which has been shown to play an essential role in regulating fibrosis, including cell proliferation and differentiation. We found that this regulatory pathway may be of importance in the AGAT/hArg metabolism, as expression of *Trpm7* was upregulated in our AGAT^-/-^ as compared to wt mice^9^.

Regarding creatine, the expression of miR-30b, miR-31, miR-130a, miR-148a, and miR-204 showed regulation towards wt levels by creatine supplementation in AGAT^-/-^ mice. MiR-30b and miR-31 were both upregulated in AGAT^-/-^ mice and found to be involved in regulating cardiomyocyte apoptosis and remodeling after myocardial injury^31,32^. MiR-148a which was highly expressed in LV tissue and showed upregulation in AGAT^-/-^ animals has been described to be involved in HF^37^, while miR-130a and miR-204 play a role in atherosclerosis. ^27,28^. Creatine levels are critical for the normally functioning heart and have been shown to be altered in CVD. Considering this, on the molecular level, the creatine-dependent regulation of CVD-related miRNAs in our AGAT^-/-^ mice may contribute to the association of creatine and cardiac dysfunction. Altogether, the observed changes in miRNA expression with creatine supplementation were more pronounced than with hArg supplementation. This matches previous findings on gene expression level, where creatine, rather than hArg supplementation, led to a normalization of gene expression levels in AGAT^-/-^ animals^9^.

Finally, by using bioinformatics target prediction, we identified potential genes regulated by the candidate miRNAs in our AGAT^-/-^ mouse model. Numerous of these genes have a well-known role in CVD and thus may provide a link for the supposed role of our candidate miRNAs for CVD. These results may inform future experimental studies to further evaluate regulatory pathways.

There are several limitations to our study. Our results must be considered hypothesis generating, as we do not provide further experimental proof of the proposed role of miRNAs in the cardiac AGAT metabolism. Interactions of miRNAs and mRNAs were predicted using bioinformatics, and experimental studies involving miRNA mimics or inhibitors are warranted to confirm interactions of specific miRNAs and target genes. Moreover, we only investigated known CVD-related miRNAs. In future projects screening of novel miRNAs shall be performed. The observed fold changes of deregulated miRNAs in our study were rather low. We hypothesize that a combination of several miRNAs with smaller effect sizes may play a role in the AGAT metabolism, overall leading to larger effect sizes. A more pronounced regulation may be present in disease models of AGAT^-/-^ mice, what would be a next step for future research. Such experiments (e.g., MI or diet fed atherosclerosis) will directly link AGAT deficiency with CVD.

In summary, our study indicates that CVD-related miRNAs play a role in the cardiac AGAT metabolism. Regulation of miRNAs and consecutive altered gene expression may represent a potential mechanism underlying the association of AGAT, hArg and creatine with cardiac (dys)function and CVD. Future experimental studies are warranted to further evaluate corresponding regulatory pathways.

## Supplementary Information

Supplementary Information.

## Data Availability

The miRNA sequencing data have been deposited in NCBI's GEO database and are accessible through the accession number GSE184723.
