# Supplementary material for: Expression of cardiovascular-related microRNAs is altered in L-arginine:glycine amidinotransferase deficient mice
Source: Sci Rep. 2022 Mar 24;12:5108. doi: 10.1038/s41598-022-08846-1 (PMC8948300; doi:10.1038/s41598-022-08846-1)
Supplement: Supplementary file 1 — Supplementary Information. [file 41598_2022_8846_MOESM1_ESM.pdf]

Supplementary Information

Expression of cardiovascular-related microRNAs is altered in L-arginine:glycine amidinotransferase deficient mice

Märit Jensen[1,2,3*], Christian Müller[2], Norbert Hübner[4,5,6], Giannino Patone[4], Kathrin Saar[4], Chi-un Choe[1,3], Edzard Schwedhelm[3,7], Tanja Zeller[2,3]

[1] Department of Neurology, University Medical Center Hamburg-Eppendorf, Hamburg, 20246, Germany

[2] University Center of Cardiovascular Science, University Heart and Vascular Center Hamburg, Department of Cardiology, University Medical Center Hamburg-Eppendorf, Hamburg, 20246, Germany

[3] German Centre for Cardiovascular Research (DZHK e.V.), partner site Hamburg/Kiel/Lübeck, Germany

[4] Cardiovascular and Metabolic Sciences, Max Delbrück Center for Molecular Medicine in the Helmholtz Association (MDC), Berlin, Germany

[5] Charité – Universitätsmedizin Berlin, corporate member of Freie Universität Berlin and Humboldt-Universität zu Berlin, Berlin, Germany

[6] German Centre for Cardiovascular Research (DZHK e.V.), partner site Berlin, Berlin, Germany

[7] Institute of Clinical Pharmacology and Toxicology, University Medical Center Hamburg-Eppendorf, Hamburg, 20246, Germany

*Corresponding author:
Märit Jensen
Department of Neurology, University Medical Center Hamburg-Eppendorf, Hamburg, 20246, Germany
m.jensen@uke.de
+49 (0) 40 7410 - 58860

Supplementary Table S1. miRNA assays (Thermo Fischer Scientific) used for qPCR analysis.

miRNA	Assay Name	Assay ID
miR-298-5p	mmu-miR-298	002598
miR-135a-5p	hsa-miR-135a	000460
miR-31-5p	mmu-miR-31	000185
miR-210 -3p	hsa-miR-210	000512
miR-30b-5p	hsa-miR-30b	000602
miR-181c-5p	hsa-miR-181c	000482
miR-148a-3p	hsa-miR-148a	000470
miR-30d-3p	hsa-miR-30d	000420
let-7i-5p	hsa-let-7i	002221
miR-130a-3p	hsa-miR-130a	000454
miR-125b-5p	hsa-miR-125b	000449
miR-204-5p	hsa-miR-204	000508
snoRNA202	snoRNA202	001232

Supplementary Table S2. Significantly regulated miRNAs between wt and AGAT$^{-/-}$ mice. False-Discovery-Rate (FDR) ≤ 0.05. FC: fold change.

miRNA	FC	P value
mir-130a-3p	1.66	1.9×10^{-29}
mir-215-5p	-1.59	1.09×10^{-14}
mir-210-3p	1.4	5.5×10^{-12}
mir-31-5p	1.5	6.4×10^{-10}
mir-5119-3p	-28.56	2.09×10^{-8}
mir-204-5p	-1.22	1.4×10^{-8}
mir-298-5p	2.05	1.2×10^{-7}
mir-470-5p	3.42	4.46×10^{-7}
mir-1983-3p	-1.95	2.23×10^{-6}
mir-125b-2-3p	1.22	2.71×10^{-6}
mir-30b-5p	1.2	3.83×10^{-6}
mir-365-1-3p	-1.45	7.84×10^{-6}
mir-365-2-3p	-1.49	2.2×10^{-5}
mir-99a-5p	1.22	2.7×10^{-5}
mir-100-5p	-1.21	3.53×10^{-5}
let-7i-5p	1.16	3.7×10^{-5}
mir-296-5p	2.06	5.12×10^{-5}
mir-1247-5p	-1.85	5.15×10^{-5}
mir-7688-5p	1.94	5.41×10^{-5}
mir-871-3p	3.1	1.02×10^{-4}
mir-210-5p	1.32	1.75×10^{-4}
mir-375-3p	2.03	1.8×10^{-4}
mir-135a-2-5p	-1.63	2.73×10^{-4}
mir-107-3p	-1.11	4.11×10^{-4}
mir-130a-5p	2.41	7.34×10^{-4}
mir-148a-3p	1.23	7.57×10^{-4}
mir-30d-3p	1.19	9.01×10^{-4}
mir-181c-5p	1.24	1.02×10^{-3}
mir-34c-5p	-1.38	1.65×10^{-3}
mir-135a-1-5p	-1.52	1.99×10^{-3}
mir-182-5p	1.39	2.07×10^{-3}
mir-499-5p	1.19	2.58×10^{-3}
mir-30d-5p	1.18	2.6×10^{-3}
